# Supplementary material for: MetaRibo-Seq measures translation in microbiomes
Source: Nat Commun. 2020 Jun 29;11:3268. doi: 10.1038/s41467-020-17081-z (PMC7324362; doi:10.1038/s41467-020-17081-z)
Supplement: Supplementary file 10 — Supplementary Data 7 [file 41467_2020_17081_MOESM10_ESM.zip › File2/Confidence_High_Taxonomy/200517_out.krona.html]

Javascript must be enabled to view this page.

members
magnitude
magnitudeUnassigned
count
unassigned
taxon
rank

200517\_out

89

89
superkingdom
2

89
phylum
976

200643
class
89

171549
order
89

1
family
171550

genus
1
239759


SRS147557\_contig\_number\_29555
28117
1
species

family
20
815

20

SRS044535\_contig\_number\_855SRS103987\_contig\_number\_13214SRS148253\_contig\_number\_13418
816
3
genus

357276

SRS048981\_contig\_number\_1569SRS075021\_contig\_number\_135
2
species

1
species
162156

SRS045004\_contig\_number\_21315

2
species
1263054

SRS104327\_contig\_number\_1249SRS142542\_contig\_number\_24660


SRS024009\_contig\_number\_8094SRS053398\_contig\_number\_145SRS097958\_contig\_number\_19996SRS104975\_contig\_number\_21553SRS144603\_contig\_number\_14834
821
5
species

47678

SRS020328\_contig\_number\_14388
1
species

1
species
585544

SRS016056\_contig\_number\_3312


SRS045713\_contig\_number\_4062
671267
1
species

310297

SRS043841\_contig\_number\_1725
species
1


SRS143372\_contig\_number\_10671
28116
1
species

818

SRS149075\_contig\_number\_9289
species
1

species
1
2292956

SRS1055069\_contig\_number\_15019

2005525
family
68

68
genus
375288

1897013

SRS014235\_contig\_number\_40943SRS143181\_contig\_number\_13528
species
2

46503

SRS011405\_contig\_number\_17592SRS013687\_contig\_number\_7317SRS013800\_contig\_number\_19490SRS013940\_contig\_number\_5024SRS014287\_contig\_number\_18373SRS014613\_contig\_number\_18088SRS014683\_contig\_number\_16618SRS015065\_contig\_number\_contig-100\_1799.206751SRS015431\_contig\_number\_82401SRS015663\_contig\_number\_27844SRS016381\_contig\_number\_2459SRS017247\_contig\_number\_8472SRS017521\_contig\_number\_46632SRS019030\_contig\_number\_11347SRS019068\_contig\_number\_108346SRS019381\_contig\_number\_19360SRS019808\_contig\_number\_9974SRS020271\_contig\_number\_1208SRS023914\_contig\_number\_498SRS024075\_contig\_number\_11697SRS024492\_contig\_number\_17087SRS042966\_contig\_number\_11498SRS045244\_contig\_number\_2892SRS045645\_contig\_number\_18875SRS046068\_contig\_number\_contig-100\_107.27574SRS048164\_contig\_number\_24267SRS049402\_contig\_number\_14344SRS049959\_contig\_number\_45614SRS051031\_contig\_number\_35000SRS051610\_contig\_number\_9086SRS052697\_contig\_number\_35828SRS053356\_contig\_number\_44258SRS053649\_contig\_number\_17198SRS062427\_contig\_number\_8385SRS063489\_contig\_number\_5702SRS064232\_contig\_number\_1127SRS064276\_contig\_number\_36546SRS064757\_contig\_number\_2963SRS075984\_contig\_number\_1293SRS077552\_contig\_number\_22017SRS077589\_contig\_number\_7821SRS098061\_contig\_number\_9947SRS098655\_contig\_number\_13178SRS101376\_contig\_number\_18473SRS1041039\_contig\_number\_2489SRS1041091\_contig\_number\_17406SRS1041092\_contig\_number\_3869SRS1041112\_contig\_number\_5354SRS1041118\_contig\_number\_3068SRS1041134\_contig\_number\_10051SRS1041137\_contig\_number\_14437SRS1041138\_contig\_number\_13435SRS1041141\_contig\_number\_8072SRS104912\_contig\_number\_10170SRS105153\_contig\_number\_26847SRS140492\_contig\_number\_25871SRS142503\_contig\_number\_21219SRS143991\_contig\_number\_17206SRS144297\_contig\_number\_9506SRS144506\_contig\_number\_26500SRS144714\_contig\_number\_29589SRS145497\_contig\_number\_4012SRS147425\_contig\_number\_7255SRS148091\_contig\_number\_4024SRS148817\_contig\_number\_4861SRS893383\_contig\_number\_26297
66
species
